# Supplementary material for: Non-Invasive Neuromodulation Methods to Alleviate Symptoms of Huntington’s Disease: A Systematic Review of the Literature
Source: J Clin Med. 2023 Mar 2;12(5):2002. doi: 10.3390/jcm12052002 (PMC10004225; doi:10.3390/jcm12052002)
Supplement: Supplementary file 1 [file jcm-12-02002-s001.zip › jcm-2220071-supplementary.pdf]

Supplementary Materials

Search strategy

The search strategy was developed with assistance of a Texas Medical Center (TMC) librarian.

Medline OVID search strategy

1. Huntington Disease/
2. ("huntington* disease*" or "huntington* chorea*").ti,ab,kw.
3. 1 or 2
4. Electric Stimulation Therapy/ or Electric Stimulation/ or Transcranial Magnetic Stimulation/ or Transcranial Direct Current Stimulation/ or Electroconvulsive Therapy/
5. (neuromodulation* or "neuro-modulation*" or "transcranial magnetic stimulation*" or "trans-cranial magnetic stimulation*" or "direct current stimulation*" or "electroconvulsive therap*" or "electro-convulsive therap*").ti,ab,kw.
6. (electric adj3 (stimulation* or brain*)).ti,ab,kw.
7. 4 or 5 or 6
8. 3 and 7

Embase search strategy

1. 'huntington chorea'/de
2. 'huntington* disease*':ti,ab,kw OR 'huntington* chorea*':ti,ab,kw
3. #1 OR #2
4. 'electrotherapy'/de
5. 'electrostimulation'/de
6. 'transcranial magnetic stimulation'/de
7. 'transcranial direct current stimulation'/de
8. 'electroconvulsive therapy'/de
9. 'neuromodulation'/de
10. neuromodulation*:ti,ab,kw OR 'neuro-modulation*':ti,ab,kw OR 'transcranial magnetic stimulation*':ti,ab,kw OR 'trans-cranial magnetic stimulation*':ti,ab,kw OR 'direct current stimulation*':ti,ab,kw OR 'electroconvulsive therap*':ti,ab,kw OR 'electro-convulsive therap*':ti,ab,kw
11. (electric NEAR/3 (stimulation* OR brain*)):ti,ab,kw
12. #4 OR #5 OR #6 OR #7 OR #8 OR #9 OR #10 OR #11
13. #3 AND #12

Cochrane search strategy

1. MeSH descriptor: [Huntington Disease] this term only
2. (("huntington* disease*" or "huntington* chorea*")):ti,ab,kw
3. #1 or #2
4. MeSH descriptor: [Electric Stimulation Therapy] this term only
5. MeSH descriptor: [Electric Stimulation] this term only
6. MeSH descriptor: [Transcranial Magnetic Stimulation] this term only
7. MeSH descriptor: [Transcranial Magnetic Stimulation] this term only
8. MeSH descriptor: [Electroconvulsive Therapy] this term only
9. ((neuromodulation* or "neuro-modulation*" or "transcranial magnetic stimulation*" or "trans-cranial magnetic stimulation*" or "direct current stimulation*" or "electroconvulsive therap*" or "electro-convulsive therap*")):ti,ab,kw
10. ((electric NEAR/3 (stimulation* or brain*))):ti,ab,kw
11. #4 or #5 or #6 or #7 or #8 or #9 or #10
12. #3 and #11

PsychInfo search strategy

1. huntingtons disease/
2. ("huntington* disease*" or "huntington* chorea*").ti,ab,id.
3. 1 or 2
4. electrical stimulation/
5. transcranial direct current stimulation/ or transcranial magnetic stimulation/
6. electroconvulsive shock therapy/
7. neuromodulation/
8. (neuromodulation* or "neuro-modulation*" or "transcranial magnetic stimulation*" or "trans-cranial magnetic stimulation*" or "direct current stimulation*" or "electroconvulsive therap*" or "electro-convulsive therap*").ti,ab,id.
9. (electric adj3 (stimulation* or brain*)).ti,ab,id.
10. 4 or 5 or 6 or 7 or 8 or 9
11. 3 and 10

Eligibility criteria	Inclusion	Exclusion
**Participants**	Patients with Huntington's disease	
**Interventions**	ECT, TMS, tDCS	Deep brain stimulation (DBS), direct electrical stimulation through surgery, functional electrical stimulation (FES), transcutaneous electrical nerve stimulation (TENS), peripheral nerve stimulation (trigeminal, supraorbital, occipital nerves), electroacupuncture, sphenopalatine ganglion stimulation (SPG), vagus nerve stimulation (VNS), pulsed radiofrequency, transcranial near-infrared stimulation (NIRS), cortical stimulation with invasive electrodes
**Comparators/Controls**	Studies that have measured the effects of neuromodulation on HD related symptoms by comparing stimulation vs. sham or standard treatment. Some studies - e.g. case reports or case series - may not have control group	
**Outcomes**	Primary (efficacy of ECT, TMS and tDCS on different symptoms (motor, cognitive and behavioral) in HD). Additional outcomes (Adherence, safety and adverse effect profiles with ECT, TMS and tDCS).	
**Study Types**	Clinical Trials, Case Reports, Case Series, Interventions- open or controlled	Screening & Diagnostic tests including Electro-physiological studies, Reviews, Experimental Studies on Animal Models, Other Systematic Reviews and Meta-Analysis

Studies classification according to Quality (Case-Report)

Authors	Were patient's demographic characteristics clearly described?	Was the patient's history clearly described and presented as a timeline?	Was the current clinical condition of the patient on presentation clearly described?	Were diagnostic tests or assessment methods and the results clearly described?	Was the intervention(s) or treatment procedure(s) clearly described?	Was the post-intervention clinical condition clearly described?	Were adverse events (harms) or unanticipated events identified and described?	Does the case report provide takeaway lessons?	Total scores
Evans et al. (1987)	Y	Y	Y	U	U	U	U	Y	4/8
Lewis et al. (1996)	Y	Y	Y	Y	Y	U	N	Y	6/8
Beale et al. (1997)	U	Y	Y	Y	Y	Y	N	Y	6/8
Merida-Puga et al. (2011)	Y	Y	Y	Y	Y	Y	U	Y	7/8
Nakano et al. (2013)	U	Y	Y	Y	N	Y	Y	Y	6/8
Magid et al. (2014)	N	U	Y	Y	U	U	U	Y	3/8
Petit et al. (2016)	U	Y	Y	Y	N	Y	U	Y	5/8
Shah et al. (2017)	U	Y	Y	Y	U	Y	N	Y	5/8
Abeysundera et al. (2019)	Y	Y	Y	Y	U	Y	U	Y	6/8
Mowfati et al. (2021)	U	Y	Y	Y	Y	Y	U	Y	6/8
Davis et al. (2016)	N	Y	Y	U	Y	Y	Y	U	5/8

Abbreviations: Y, Yes; N, No; U, Unclear; N/A, Not Applicable.

Studies classification according to Quality (Case-Series)

Authors	Were there clear criteria for inclusion in the case series?	Was the condition measured in a standard, reliable way for all participants included in the case series?	Were valid methods used for identification of the condition for all participants included in the case series?	Did the case series have consecutive inclusion of participants?	Did the case series have complete inclusion of participants?	Was there clear reporting of the demographics of the participants in the study?	Was there clear reporting of clinical information of the participants?	Were the outcomes or follow up results of cases clearly reported?	Was there clear reporting of the presenting site(s)/clinic(s) demographic information?	Was statistical analysis appropriate?	Total scores
Ranen et al. (1994)	U	Y	Y	U	Y	Y	Y	Y	Y	N/A	7/10
Cusin et al. (2013)	Y	Y	U	Y	Y	N	Y	Y	Y	N/A	7/10
Adrissi et al. (2019)	Y	Y	Y	Y	Y	Y	Y	Y	Y	N/A	9/10
Shukla et al. (2013)	U	Y	U	N	N	N	Y	Y	N	N/A	3/10

Studies classification according to Quality (RCTs)

Authors	Was true randomization used for assignment of participants to treatment groups?	Was allocation to treatment groups concealed?	Were treatment groups similar at the baseline?	Were participants blind to treatment assignment?	Were those delivering treatment blind to treatment assignment?	Were outcomes assessors blind to treatment assignment?	Were treatment groups treated identically other than the intervention of interest?	Was follow up complete and if not, were differences between groups in terms of their follow up adequately described and analyzed?	Were participants analyzed in the groups to which they were randomized?	Were outcomes measured in the same way for treatment groups?	Were outcomes measured in a reliable way?	Was appropriate statistical analysis used?	Was the trial design appropriate, and any deviations from the standard RCT design (individual randomization, parallel groups) accounted for in the conduct and analysis of the trial?	Total scores
Brusa et al. (2005)	N	U	Y	U	U	Y	Y	U	U	Y	Y	Y	N	6/13
Groiss et al. (2012)	U	U	Y	Y	U	Y	Y	Y	Y	Y	Y	Y	Y	10/13
Eddy et al. (2017)	Y	Y	Y	Y	U	Y	Y	Y	Y	Y	Y	Y	Y	12/13
Bocci et al. (2020)	U	U	Y	Y	U	Y	Y	U	Y	Y	Y	Y	Y	9/13
